# Protective Effects of L-Theanine on IPEC-J2 Cells Growth Inhibition Induced by Dextran Sulfate Sodium via p53 Signaling Pathway

**DOI:** 10.3390/molecules26227002

**Published:** 2021-11-19

**Authors:** Longlin Zhang, Mengmeng Ma, Zhengyi Li, Haihan Zhang, Xi He, Zehe Song

**Affiliations:** 1 Department of Animal Science, College of Animal Science and Technology, Hunan Agricultural University, Changsha 410128, China; zllwithann@gmail.com (L.Z.); lonnie_970205@126.com (M.M.); lzy1054337321@163.com (Z.L.); zhhous@163.com (H.Z.); hexi111@126.com (X.H.); 2 Hunan Co-Innovation Center of Animal Production Safety, Hunan Agricultural University, Changsha 410128, China

**Keywords:** L-theanine, proliferation, IPEC-J2 cells, metabolomics

## Abstract

L-theanine is a nonprotein amino acid found in tea leaves and has been widely used as a safe food additive in beverages or foods because of its varied bioactivities. The aim of this study was to reveal the in vitro gastrointestinal protective effects of L-theanine in DSS-induced intestinal porcine enterocyte (IPEC-J2) cell models using molecular and metabolic methods. Results showed that 2.5% dextran sulfate sodium (DSS) treatment inhibited the cell proliferation of IPEC-J2 and blocked the normal operation of the cell cycle, while L-theanine pretreatment significantly preserved these trends to exert protective effects. L-theanine pre-treatment also up-regulated the EGF, CDC2, FGF2, Rb genes and down-regulated p53, p21 proliferation-related mRNA expression in DSS-treated cells, in accompany with p53 signaling pathway inhibition. Meanwhile, metabolomics analysis revealed that L-theanine and DSS treated IPEC-J2 cells have different metabolomic profiles, with significant changes in the key metabolites involved in pyrimidine metabolism and amino acid metabolism, which play an important role in nucleotide metabolism. In summary, L-theanine has a beneficial protection in DSS-induced IPEC-J2 cells via promoting proliferation and regulating metabolism disorders.

## 1. Introduction

Weaning stress, an important factor restricting pig production, will reduce feed intake and slow down the growth rate of weaned piglets, which has a great impact on the efficiency of pig production. As reported, the main target of weaning stress is the intestinal tract, especially the small intestine, in which the morphology and structure of its damage are particularly serious [1]. Numerous studies have shown that within 3 days of weaning, the relative weight of small intestinal tissue and mucosa decreased in piglets, with atrophy of the villus height and proliferation of the crypt [1,2,3]. These changes were caused by the increased rate of apoptosis and decreased rate of cell proliferation. As we know, the precise balance of the epithelial cell proliferation and apoptosis is necessary to maintain epithelial integrity. Furthermore, it is the key of the physical barrier, which regulates microbial colonization and prevents them from infiltrating the epithelium [4,5]. Previous studies have shown that weaning stress was often accompanied by intestinal barrier damage, resulting in secondary intestinal function damage and dysfunction. However, the balance mechanism of epithelial cell proliferation and apoptosis in it remains largely unexplored.

Plant extracts are always considered as an attractive approach for the remission of weaning stress [6,7,8]. L-theanine (γ-glutamylethylamide), a glutamate derivative, is a nonprotein amino acid found in tea leaves and has been widely used as a safe food additive in beverages or foods because of its varied bioactivities, such as relaxation, mental concentration, increased sleep quality, antiapoptotic, antioxidant and anti-inflammatory etc. [9,10,11]. Meanwhile, it has been reported that the anti-apoptotic and protective effects of L-theanine on inflammatory injuries in the brain, stomach and liver [9,10]. However, the potential of L-theanine for the treatment of anti-apoptotic in the intestine has not been comprehensively investigated.

In this study, the protective effect of L-theanine on DSS-induced IPEC-J2 cells is shown, with a highlight on the regulation of cell proliferation as well as the inactivation of the p53 signaling pathway. Furthermore, the metabolomics analysis by UPLC-Q-TOF/MS was performed, revealing the regulatory effect of L-theanine on protecting cellular metabolic pathways against DSS-induced IPEC-J2 cell metabolism disorders.

## 2. Results

### 2.1. Dose-Effect of L-Theanine on the Viability of IPEC-J2 Cells

**Figure 1:**
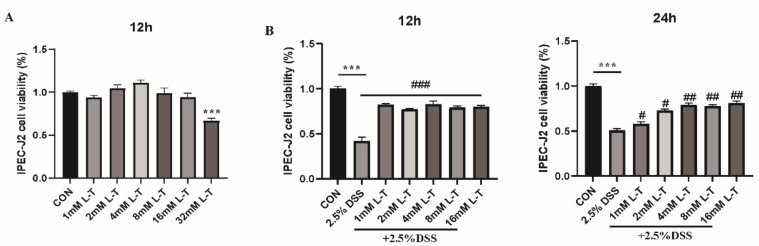
Effect of L-theanine on IPEC-J2 cells viability with/without DSS induction. (**A**) Cells were treated with the indicated concentrations of L-theanine for 12 h. *** *P* < 0.001, versus the CON group without L-theanine treatment; (**B**) Cells were pretreated with the indicated concentrations of L-theanine for 2 h and then stimulated with 2.5% DSS for 12 h and 24 h. The DSS group was obtained in the absence of L-theanine. *** *P* < 0.001, compared with the CON group. # *P* < 0.05, ## *P* < 0.01 and ### *P* < 0.001 versus DSS group. CON, DMEM without DSS and L-theanine; L-T, DMEM contained L-theanine; DSS, DMEM contained DSS.

Effective concentrations of L-theanine (0–32 mM) were evaluated by pretreatment of IPEC-J2 cells for 12 h. The result showed that there was no inhibitory effect on cells growth with the treatment of L-theanine below 16 mM, while the L-theanine above 32 mM exhibited notable inhibition of cell viability compared to the control group (*P* < 0.01) (Figure 1A). Therefore, the L-theanine concentration range of 0–16 mM was used in further experiments.

### 2.2. Effect of L-Theanine on DSS-Induced IPEC-J2 Cells

As shown in Figure 1B, the effect of 2 h pretreatment with assigned concentrations of L-theanine (0–16 mM) on the viability of IPEC-J2 cells induced by 2.5% DSS (12 h and 24 h) was evaluated. When treated with DSS alone, the viability of IPEC-J2 cells was significantly reduced below 50% (*P* < 0.01). However, a noticeable promotion of cell growth with L-theanine pretreatment compared to the DSS group was observed (*P* < 0.05). Therefore, 4 mM of L-theanine was designated for further evaluation of its protective efficacy on IPEC-J2 cells against DSS-induced damages.

### 2.3. Effect of L-Theanine on the Proliferation and Apoptosis of IPEC-J2 Cells Induced by DSS

**Figure 2:**
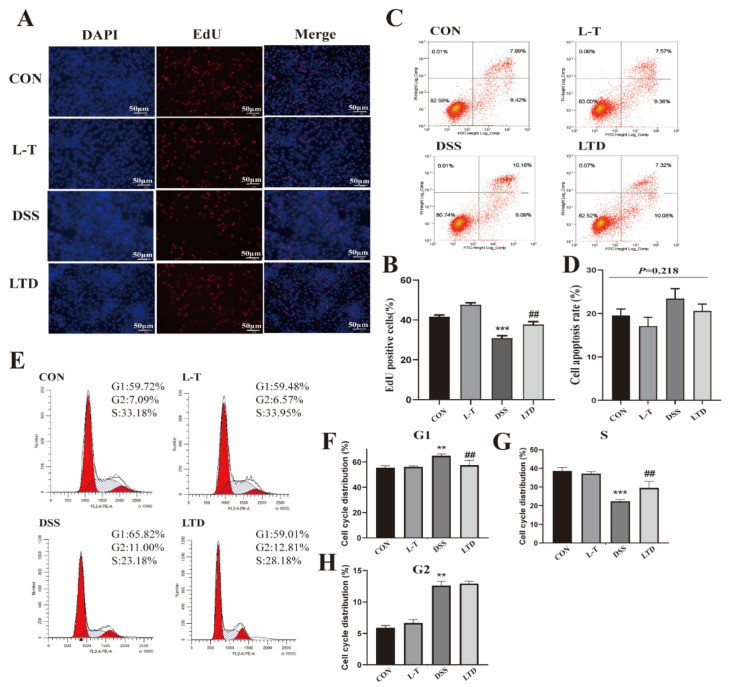
Effect of L-theanine on the proliferation and apoptosis of IPEC-J2 cells induced by DSS. (**A**,**B**) Representative images of EdU staining of IPEC-J2 cells and results of the analysis of EdU-positive cells (*n* = 4) treated with DSS and L-theanine. Scale bar = 50 μm. (**C**,**D**) Results of cell apoptosis in four groups analyzed by FACSCanto II flow cytometer (*n* = 4). (**E**–**H**) G1, DNA-presynthetic phase; G2, DNA-postsynthetic phase; S, DNA-synthesis phase. Results of cell cycle in four groups analyzed by FACSCanto II flow cytometer (*n* = 4). ** *P* < 0.01 and *** *P* < 0.001, compared with the CON group. ## *P* < 0.01 versus DSS group.

**Figure 3:**
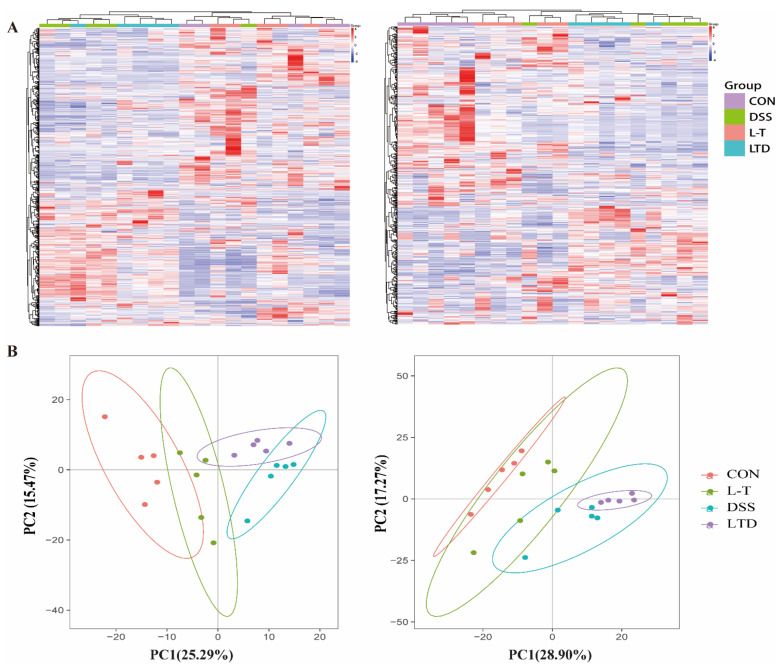
Hierarchical clustering analysis (HCA) (**A**) and principal component analysis (PCA) (**B**) of the differences on cellular metabolites among CON, L-T, DSS and LTD groups (*n* = 5) based on mass spectrometry data acquired in both positive and negative ionization modes.

Treatment with DSS showed an upward trend but did not significantly affect the apoptosis of IPEC-J2 cells compared to the other groups (*P* > 0.1) (Figure 2C,D). In order to explore the reason for the changing of cell viability (Figure 1B), the cell cycle and proliferation of cells were assayed. As shown in Figure 3E–H, the cell cycle analysis revealed that DSS significantly increased the percentage of cells in the G1 and G2 phase, while significantly decreasing the percentage of cells in the S phase compared with the control group (*P* < 0.05). Furthermore, L-theanine 2-h pretreatment significantly reversed these trends (*P* < 0.05), except for the percentage of cells in the G2 phase (*P* > 0.1). Similarly, the EdU incorporation assay result showed that DSS exhibited significantly lower mitotic activity than the control group (*P* < 0.01), whereas the mitotic activity in the LTD group was significantly promoted (*P* < 0.05) (Figure 2A,B). These results suggest that L-theanine might promote the cell cycle progression and cell proliferation of IPEC-J2 cells induced by DSS.

### 2.4. Effect of L-Theanine on DSS-Induced IPEC-J2 Cells Metabolism Disorders

#### 2.4.1. Cellular Metabolites Detection and Analysis

DSS caused severe damage by inhibition of cell proliferation and cell cycle, which lead to disturbances in cell metabolism, and L-theanine played the protective role by reversion. In order to explain this, the UPLC-Q-TOF/MS method for the detection and analysis of cell metabolites was used. The result showed different levels of metabolites in the CON, L-T, DSS, and LTD groups. The metabolites in the LTD group were relatively nearer to the CON and L-T groups than the DSS group by HCA analysis and PCA analysis (Figure 3A,B). In addition, DSS also led to more down-regulated metabolites (139 total up-regulated and 202 total down-regulated), while L-theanine pretreatment can up-regulate more metabolites (89 total up-regulated and 47 total down-regulated) (Table 1). This finding indicated that the pre-treated L-theanine may prefer to up-regulate the metabolite expression to deal with the cell metabolic disorder caused by the down-regulated metabolite expression of DSS. Furthermore, we screened 88 metabolites with *P*-values < 0.05 and fold changes >2, and the results showed significant differences of biomarkers in the CON, DSS and LTD groups (Table 2). Furthermore, we found cytidine and LPA (20:1) were the common metabolites with the opposite regulated trend, which might be the regulatory target of L-theanine. These findings indicated that the pretreated L-theanine participated in regulating the cell metabolism close to a relative normal condition against DSS induction.

#### 2.4.2. Pathway Analysis

**Figure 4:**
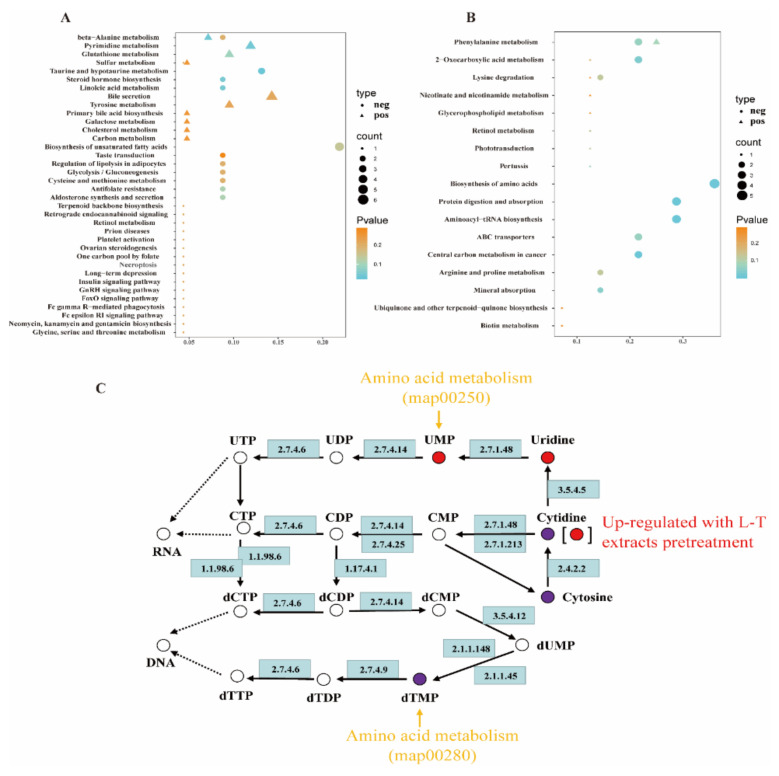
Analysis of the global metabolic pathways with L-theanine and DSS. (**A**,**B**) The metabolic pathways enrichment results of the DSS group compared to the control group and LTD group compared to the DSS group with *P*-value < 0.3. (**C**) The regulated biomarkers during Pyrimidine metabolism. Cytidine, cytosine, and dTMP (purple point) were down-regulated in the DSS group compared to the CON group. Cytidine, UMP and Uridine (red point) were up-regulated in LTD group compared to DSS group. UMP and dTMP (yellow arrow) are the metabolisms of the amino acids of L-theanine treatment regulation. Different numbers in the blue background indicate different enzymes (refer to KEGG pathway analysis).

To further explore the metabolic mechanism, the global metabolic pathways analysis was preceded in this study. The metabolic pathways enrichment of differential metabolites was performed; when the ratio was satisfied by x/y (number of differential metabolites in the corresponding metabolic pathway/number of total metabolites identified in the pathway), metabolic pathways were considered as enrichment. The metabolic pathway enrichment results of the DSS group compared to the control and LTD groups compared to the DSS group are shown in Figure 4A,B with *P*-values < 0.3. Furthermore, six metabolic pathway enrichments in the DSS group compared to the control group and 10 metabolic pathway enrichments in the LTD group compared to the DSS group were screened with *P*-values < 0.05, the details of which are listed in Table 3. Above all, we can see that pyrimidine metabolism had the highest number of total compounds and biomarkers in the DSS group compared to the control group, while the biosynthesis of amino acids was the highest in the LTD group compared to the DSS group. It indicated that the pyrimidine metabolism was the most significant change during metabolism regulation by DSS-induced damage, while L-theanine mainly played a protective role in response to DSS injury through amino acid metabolism (Figure 4C).

### 2.5. Effect of L-Theanine on the Proliferation-Related Genes Expression of IPEC-J2 Cells Induced by DSS

**Figure 5:**
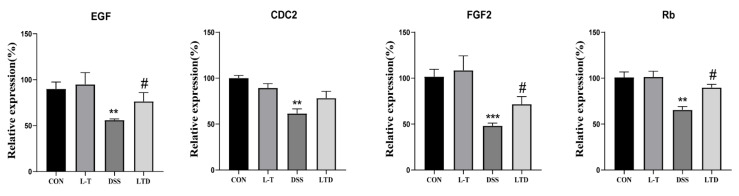
Effect of L-theanine on the proliferation-related genes expression of IPEC-J2 cells induced by DSS. The mRNA relative expression of the cell cycle-related genes EGF, CDC2, FGF2, and Rb were detected using RT-PCR assay (*n* = 6). ** *P* < 0.01 and *** *P* < 0.001, compared with the CON group. # *P* < 0.05.

In order to explore the mechanism by which L-theanine promotes cell cycle and proliferation, the expression of genes associated with proliferation was measured. As shown in Figure 5, compared with the CON group, the DSS group was significantly decreased the epidermal growth factor (EGF), cell division cycle (CDC2), fibroblast growth factor (FGF2), retinoblastoma gene (Rb) mRNA expression, while the LTD group significantly reversed these trends except in the CDC2 (*P* < 0.05).

### 2.6. Effect of L-Theanine on the p53 Cell Signaling Pathway of IPEC-J2 Cells Induced by DSS

**Figure 6:**
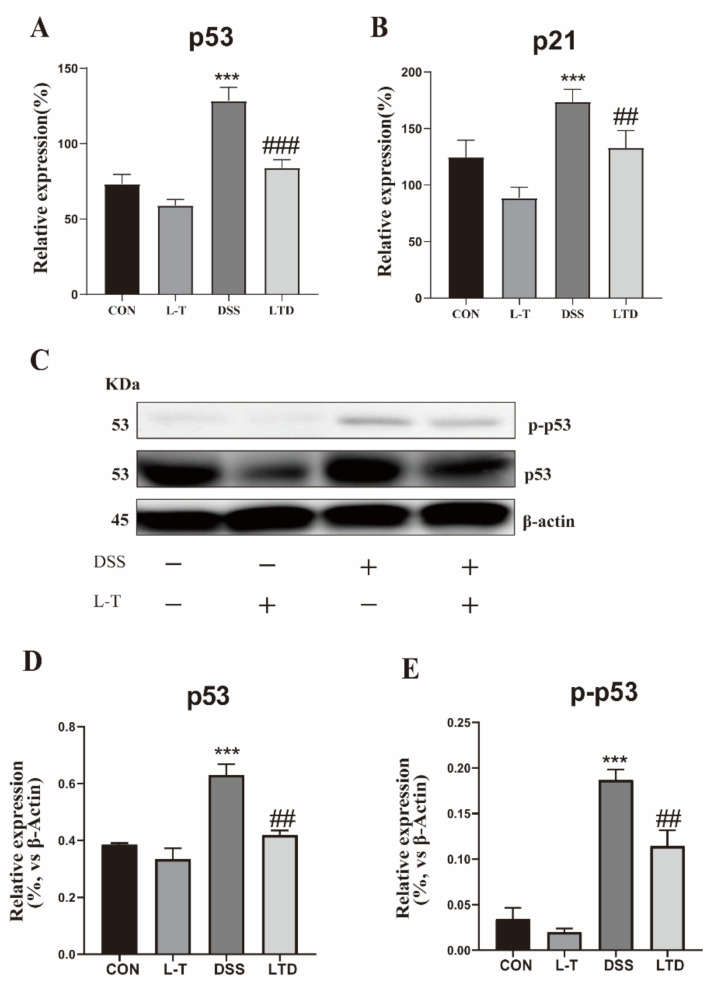
Effect of L-theanine on p53 signaling pathway of IPEC-J2 cells induced by DSS. (**A**,**B**) The mRNA relative expression of p53 and p21 genes were detected using RT-PCR assay (*n* = 6). (**C**–**E**) The protein relative expression of p53 and p-p53 were detected using Western blotting assay (*n* = 3). ** *P* < 0.01 and *** *P* < 0.001, compared with the CON group. ## *P* < 0.01 and ### *P* < 0.001 versus DSS group.

Tumor-suppressor p53 has an important role in cell proliferation. In this study, 2.5% DSS treatment increased the p53 and p21 mRNA expression, while the LTD group significantly reversed these trends (Figure 6A,B). Protein expression showed that DSS led to acutely increased p53 and p-p53, while L-theanine 2-h pretreatment recovered its up-regulation effects on all proteins (Figure 6C–E). These results suggest that L-theanine can block the p53 signaling pathway to promote cell cycle progression and cell proliferation of IPEC-J2 cells induced by DSS.

## 3. Discussion

Weaning stress has always been one of the important factors affecting the healthy growth of piglets. Due to the changes in food, social and living environments, the morphology, physiological function and intestinal microbe of weaned piglets changed significantly, especially intestinal barrier damage and intestinal inflammation [2]. Because the histological features, clinical manifestations, site of occurrence, and cytokine proliferation of the DSS-induced colitis are very similar to that of intestinal inflammation, DSS is widely used in inflammation-modeling studies in piglets by researchers [6,12,13]. As a characteristic amino acid extracted from tea, L-theanine can be absorbed and metabolized by the small intestine [11]. However, the protective effects of L-theanine on intestinal inflammation are rarely reported, and the mechanism is hardly studied. Thus, we had decided to confirm the effects of L-theanine in DSS-induced IPEC-J2 cells. The results of the present study showed that the cell viability of DSS-induced cells was increased remarkably in L-theanine pre-treatment, although L-theanine treatment alone had no significant effects except a high concentration of toxicity. This suggests that L-theanine has a better therapeutic effect when cells are damaged, but alone may not have any obvious effect in low concentrations, and it needs to be added in moderation, as high concentrations may be harmful to the body. Meanwhile, it also suggests that L-theanine exerts different effects under normal and DSS stimulation conditions, which may also explain the health care effects of L-theanine in inflammatory bowel diseases (IBDs).

Many studies reported that intestinal barrier disruption is the hallmark of intestinal inflammation [5,14,15]. Furthermore, the precise balance of the epithelial cell proliferation and death is necessary to maintain epithelial integrity. Apoptosis and proliferation are also considered to be the direct and important causes of the changes in cell viability. Various researches have discussed the anti-apoptosis mechanisms of L-theanine [9,16]. However, whether L-theanine can prevent intestinal cell apoptosis remains unclear yet. It is also unclear that whether DSS damages intestinal cells via apoptosis. Our results showed that L-theanine and DSS tends to affect apoptosis, but not significantly. Moreover, we found that 2.5% DSS treatment significantly decreased the percentage of EdU positive cells and proliferation-related (EGF, CDC2, FGF2, Rb) genes expression, and L-theanine pretreatment can reverse these trends. Consistent with our study, Dias et al. reported that exposure of hSCs to 50 µM of L-theanine also increased cell proliferation [17]. These results suggest that the mechanism of the DSS-damaged model might be inhibiting cell proliferation, while L-theanine pretreatment can promote it.

Cell cycle, closely related to proliferation, is an important regulatory factor and evaluation parameter of body metabolism [18,19]. Furthermore, the intestinal epithelium cells have a frequent cell cycle; therefore, cell cycle arrest may lead to breaks in the barrier and absorbtion functions. However, it is still unclear whether treatment with L-theanine in DSS-induced cells is involved in the cell cycle arrests. Therefore, we evaluated the effects of L-theanine and DSS on the cell cycle. The process of the cell cycle is complex and rigorous, which involves the doubling of DNA and other cellular contents to duplicate a cell [20,21]. The cell cycle is divided into four phases, including the G1, S, G2, and M phases. The mechanism of cell entry checkpoints determines whether the cell will proceed to division or stop [12,13]. Once the cell cycle has abnormalities, the checkpoint signaling pathway is activated, immediately preventing the cell cycle to ensure the validity of DNA replication and chromosome division [22,23]. As reported, it was well-known that the cell cycle arrested cells were into apoptosis through p53 and p21 [14]. Additionally, it was also reported that DSS provoked necroptosis in the colons of DSS-treated mice [15]. Therefore, the mechanism of DSS-induced colitis is not particularly clear. In our study, after DSS treatment, the proportion of G1 phase cells significantly increased and S phase significantly decreased, which was the opposite in L-theanine pretreatment. Similar with our findings, many studies have reported that DSS essentially induced cell cycle arrest, particularly decreasing proliferative cells (-G1-SG2-M-G2-) [24,25,26,27].

Furthermore, we used the UPLC-Q-TOF/MS method for the detection and analysis of cell metabolites. The result showed different levels of metabolites in the CON, L-T, DSS, and LTD groups. Furthermore, the pyrimidine metabolism was the most significant change during metabolism regulation by DSS-induced damage, while L-T mainly played a protective role in response to DSS injury through amino acid metabolism. Cytidine as the common metabolite had the opposite regulating trend in the DSS and LTD group, which indicated it might also be a regulatory target of L-theanine. Furthermore, it is a pyrimidine nucleoside comprised of a cytosine bound to ribose via a beta-N1-glycosidic bond. As reported, cytarabine is a cell cycle-specific drug that inhibits cell division by inhibiting DNA synthesis during the S phase of the cell cycle, which is an analogue of cytidine—the main component of DNA [19]. Those results indicated that DSS caused DNA damage and triggered the G1-S-phase arrest, which lead to disturbances in cell metabolism, while L-theanine pretreatment can reverse this.

In previous studies, p53 is regarded as a major checkpoint protein in mammalian cells [20,21], and plays a critical role in sensing genotoxic and other stresses, such as DNA damage, that could potentially alter the genetic material of cells and alert the checkpoint proteins and halt cell growth and proliferation [28]. In our study, DSS treatment increased p53 and p-p53, while L-theanine 2h pretreatment recovered its regulation effects. This suggested that the P53 signaling pathway is involved in the G1-S phase arrest induced by DNA damage after DSS treatment, which inhibits cell proliferation.

Taken together, we demonstrated the protective effects of L-theanine on DSS-induced IPEC-J2 cells. The results showed that DSS caused severe damage by inhibition of cell proliferation and cell cycle via the p53 pathway, which lead to disturbances in cell metabolism, and L-theanine played a protective role by reversion. It provides an experiment-based reference for the usage of L-theanine in the feeds and food industry to relieve weaning stress in piglets.

## 4. Materials and Methods

### 4.1. Materials

L-theanine (CAS#3081-61-6, >99% purity) was obtained from Yuanye Bio-Technology, Inc. (Shanghai, China). DSS (MW 36,000–50,000) was purchased from Coolaber Science & Techenology, Inc. (Beijing, China).

### 4.2. Cell Culture and Viability

**Figure 7:**
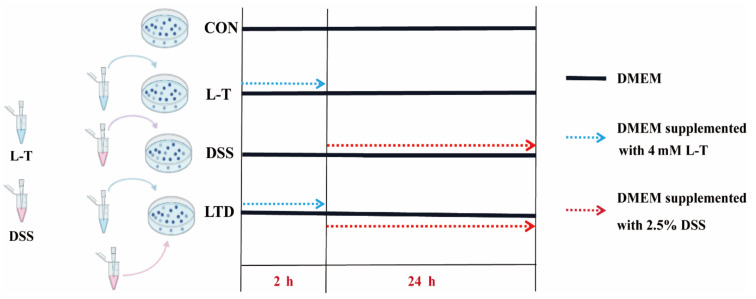
The test design of four treatments in this study according to the results of the CCK-8 assay.

The IPEC-J2 cells were obtained from the Institute of Subtropical Agriculture (Changsha, China). Cells were maintained in DMEM/F12 medium (BI, Dibosi Biological Technology, Co., Ltd., Shanghai, China), supplemented with 100 U/mL penicillin, 100 μg/mL streptomycin, and 10% (*vol/vol*) heat-inactivated fetal bovine serum (FBS) (Gibco, Carlsbad, CA, USA), at 37 °C and 5% CO_2_ in a humidified incubator. Cell viability was examined by cell counting kit-8 assay (CCK-8, Dojindo, Kumamoto, Japan) using a multi-mode microplate reader system (SpectraMax^®^ i3 Platform from Molecular Devices, Graz, Austria). According to the results of the CCK-8 assay, the design of four treatments in this study is shown in Figure 7.

### 4.3. Cell Proliferation Assay

IPEC-J2 cells were seeded in a 96-well culture plate at a density of 1 × 10^4^ cells/well in 100 μL of culture medium. For the EdU assay, 100 μL of EdU medium (50 μmol) was added to each well 24 h after transfection, and cells were incubated for 2 h at 37 °C. Then, DNA staining solution and EdU staining solution were added to each well to mark living cells (blue) and the proliferating (red) cells according to the manufacturer’s protocols. The fluorescence microscope was used to observe the cells at 20× and ImageJ software was used to determine cell numbers. At least three independent biological replicates were used in this assay.

### 4.4. Cell Apoptosis Assay

Cells were seeded in a 6-well culture plate with 2 mL culture medium. Cell apoptosis was detected using an Annexin V-FITC apoptosis detection kit (Nanjing KeyGen Biotech, China). After four-group treatment, cells were collected in a 1.5 mL centrifuge tube. Before Annexin V-FITC apoptosis analysis, cells were washed three times and double-stained with FITC-Annexin V and propidium iodide (PI). Then, cell samples were analyzed using a FACSCanto II Flow Cytometer (Becton Dickinson, Franklin Lakes, NJ, USA). Percentages of early apoptosis and late apoptosis cells were counted and used as the cell apoptosis rate. Four independent replicates were conducted for each group.

### 4.5. Cell Cycle Assay

Cells were seeded in a 6-well culture plate with 2 mL culture medium. The cell cycle assay was analyzed using a cell cycle testing kit (Nanjing KeyGen Biotech, China) according to the manufacturer’s protocols. After four-group treatment, cells were washed three times using PBS and harvested in a 1.5 mL centrifuge tube. Then, cells were incubated in 70% (*v/v*) ethanol overnight at −20 °C and then in PI solution (50 mg/mL) for 30 min at 4 °C. The cell suspension was analyzed on a FACSCanto II flow cytometer (Becton Dickinson, Franklin Lakes, NJ, USA). Four independent replicates were conducted for each group.

### 4.6. Real-Time qPCR

Total RNA was collected and extracted using the RNA Pure Kit (Aidlab Biotechnologies Co., Ltd., Beijing, China). RNA reverse transcription was performed with PrimeScript RT Reagent Kit (Accurate Biotechnology Co., Ltd.). Quantitative real-time PCR was implemented using Bioer LineGene 9600 system (Bioer Technology, Hangzhou, China) with the SYBR premix EX Taq (TaKaRa, Dalian, China) as described previously [22]. The reaction conditions were as follows: initial incubation step of 5 min at 65 °C, reverse transcription of 40 min at 37 °C and 70 °C for 10 min, followed by 45 cycles of 15 s at 95 °C for denaturation, and 60 s at 58 °C for annealing and extension. The gene-specific primers of selected cytokines are listed in Table 4. The expression of housekeeping gene GAPDH was used to normalize the expression levels of these target genes, and the relative levels of target genes were calculated using the 2^−ΔΔCt^ method.

### 4.7. Western Blotting

Western blotting analysis was performed as previously described [23]. Briefly, cells were harvested and lysed using protease inhibitor and RIPA buffer (1:100) (Beyotime, Shanghai, China). Furthermore, the protein concentration was measured using a bicinchoninic acid protein assay kit (BCA) (Beyotime, Shanghai, China) according the manufacturer’s protocols. The boiled protein samples were electrophoresed on 10% SDS-polyacrylamide gels and then transferred onto a PVDF membrane (Beyotime, Shanghai, China). The membrane containing protein fractions was blocked with 5% non-fat milk for 2 h and incubated with p53(1:1000 dilution; Proteintech, Chicago, Illinois, USA), p-p53(1:1000 dilution; CST, Boston, MA, USA) and β-actin overnight at 4 °C. After washing, the membrane was incubated with secondary antibodies (1 μg/L, Proteintech, Chicago, Illinois, USA) for 2 h at room temperature. Protein bands were visualized using an ECL advanced Western blotting detection kit (Beyotime, Shanghai, China). The relative amounts of proteins associated with specific antibodies was quantified with Lumi Vision imager software (TAITEC Co, Saitama, Japan).

### 4.8. Extraction of Cell Metabolites

The cell metabolites analysis was performed as previously described [29]. Briefly, the cells were washed using pre-cold PBS three times gently, and added 2 mL of 80% (*vol/vol*) methanol (pre-chilled to −80 °C). The plate was incubated at −80 °C for 1 h before scraping the cells from the plate, transferring them to a sterile tube. Another 1 mL of 80% methanol was added to the plate and then the mixture was thoroughly transferred to the same tube, followed by centrifugation at 14,000 g for 20 min at 4–8 °C. The supernatant was collected in a new tube and dried under the protection of nitrogen. All the operations were processed on dry ice. The dried extract was re-dissolved in 80% methanol and filtered using a 0.22 μm millipore filter (Nylon 66, Jinteng, Tianjin, China) before analyzing by UPLC-Q-TOF/MS.

### 4.9. Statistical Analysis

SPSS version 26.0 was used to perform one-way ANOVA analysis. The value of *P* < 0.05 was considered statistically significant. All trials were performed in triplicate. Data were presented as mean ± SEM. In addition, hierarchical clustering analysis (HCA) and principal component analysis (PCA) were performed by R software (v4.1.1). The functions of these metabolites and metabolic pathways were studied using the KEGG database.

## Figures and Tables

**Table 1:** The number of metabolites in CON, DSS and LTD groups.

Iteam	Number of Total Metabolites	Number of Differential Metabolites	Up	Down	Ionization Mode
DSS VS CON	767	202	106	96	(+)
	498	139	33	106	(−)
LTD VS DSS	767	76	36	40	(+)
	498	60	53	7	(−)

**Table 2:** The list of biomarkers in CON, DSS and LTD groups (*P* value < 0.05 and fold change >2).

Name	Kegg_ID	*P* Value (CVA) ^a^	Up/Down	*P* Value (DVC) ^b^	Up/Down	Ionization Mode
Penicillin G	C05551	0.0034	down			(+)
d-Alanyl-d-alanine	C00993	0.0176	up			(+)
Coniferyl alcohol	C00590	0.0205	up			(+)
Naringenin	C00509			0.0009	down	(+)
Mevalonic acid	C00418	0.0402	up			(+)
Vitamin A	C00473	0.0009	down			(+)
Retinal	C00376			0.0133	down	(+)
Desthiobiotin	C01909					(+)
Nicotinic acid	C00253			0.0396	down	(+)
Triethanolamine	C06771			0.0222	down	(+)
Pyroglutamic acid	C01879	0.0074	down			(+)
L-Pyroglutamic acid	C01879	0.0202	down			(+)
Pantothenic acid	C00864	0.0002	down			(+)
L-Kynurenine	C00328	0.0063	up			(+)
Serotonin	C00780	0.0032	up			(+)
Indole-3-acetic acid	C00954	0.0032	down			(+)
Phenylpyruvic Acid	C00166			0.0038	down	(+)
Phenylacetylglycine	C05598	0.0019	down			(+)
2-Phenylacetamide	C02505			0.0099	down	(+)
Hippuric acid	C01586	0.0041	down			(+)
3-Methoxytyramine	C05587	0.0064	up			(+)
(R)-(-)-Epinephrine	C00788	0.0029	down			(+)
Methylimidazoleacetic acid	C05828	0.0032	down			(+)
4-Guanidinobutyric acid	C01035	0.0434	down			(+)
Spermine	C00750	0.0010	up			(+)
Spermidine	C00315	0.0000	up			(+)
*cis*-4-Hydroxy-d-proline	C03440	0.0002	down			(+)
L-Saccharopine	C00449	0.0005	down			(+)
Pipecolic acid	C00408	0.0017	up			(+)
N6-Acetyl-L-lysine	C02727			0.0121	up	(+)
L-Cystathionine	C02291	0.0059	up			(+)
O-Phospho-L-serine	C01005	0.0000	down			(+)
Serine	C00065	0.0003	up			(+)
L-Asparagine	C00152	0.0018	up			(+)
D-Glucosamine 6-phosphate	C00352	0.0000	down			(+)
dTMP	C00364	0.0029	down			(+)
Cytidine	C00475	0.0407	down	0.0217	up	(+)
Cytosine	C00380	0.0003	down			(+)
Uridine 5’-monophosphate	C00105	0.0019	down			(+)
Pseudouridine	C02067	0.0146	up			(+)
Deoxyguanosine	C00330	0.0022	up			(+)
Adenine	C00147	0.0059	down			(+)
Xanthine	C00385	0.0051	down			(+)
17alpha-Hydroxyprogesterone	C01176	0.0016	down			(+)
p-Coumaric acid	C00811	0.0003	up			(+)
Glycocholic acid	C01921	0.0005	up			(+)
Taurocholic acid	C05122	0.0042	up			(+)
D-Galactosamine	C02262	0.0009	up			(+)
N-Acetyl-d-galactosamine	C01132	0.0488	down			(+)
Succinic Acid	C00042	0.0193	down			(+)
Dodecanedioic acid	C03990	0.0062	down			(−)
N-Acetylmannosamine	C16513	0.0084	down			(−)
N-Acetyl-L-phenylalanine	C06429	0.0272	down			(−)
1,3-Dimethyluracil	C06428	0.0117	down			(−)
19-Nortestosterone	C00486			0.0108	up	(−)
Dodecanoic acid	C02191	0.0206	up			(−)
Pentadecanoic acid	C00931					(−)
Prostaglandin A3	C16683	0.0067	down			(−)
LPC 16:1	C00415	0.0007	down			(−)
PMeOH (16:0–18:1)	C01182	0.0348	down			(−)
Phosphoribosyl pyrophosphate	C01595	0.0077	down			(−)
FAHFA (18:1/22:3)	C00219	0.0171	down			(−)
LPA 20:1	C14179	0.0117	up	0.0052	down	(−)
2’-Deoxyadenosine 5’-monophosphate (dAMP)	C00805	0.0017	down			(−)
L-Histidine	C00601			0.0001	up	(−)
Flavin mononucleotide (FMN)	C03519			0.0233	up	(−)
Adenosine 3’5’-cyclic monophosphate	C00135			0.0029	up	(−)
Υ-Aminobutyric acid (GABA)	C00148			0.0322	up	(−)
LPS 20:4	C00047			0.0132	up	(−)
Uridine monophosphate (UMP)	C00431			0.0004	up	(−)
Kynurenic acid	C00021	0.0020	down			(−)
D-(+)-Malic acid	C00073			0.0005	up	(−)
Salicylic acid	C00460			0.0262	down	(−)
Cer-EODS (d21:0/15:0-O-18:1)	C00295	0.0000	down			(−)
2,4-Dinitrophenol	C07130	0.0023	up			(−)
MAM2201 N-pentanoic acid metabolite	C00130	0.0088	up			(−)
Uridine	C00437			0.0004	up	(−)
Uridine 5’-diphosphoglucuronic acid	C00049	0.0000	up			(−)
L-Tyrosinemethylester	C02140	0.0063	down			(−)
1-acetyl-N-(6-chloro-1,3-benzothiazol-2-yl)-4-piperidinecarboxamide	C03681	0.0054	down			(−)
Pyrrole-2-carboxylic acid	C00353	0.0002	up			(−)
DL-Malic acid	C00423			0.0107	up	(−)
ent-Prostaglandin F2α	C00245	0.0007	up			(−)
Heneicosanoic acid	C06423			0.0113	down	(−)
Ethyl paraben	C00167			0.0206	up	(−)
Phenylacetaldehyde	C00031	0.0001	up			(−)
D-(-)-Mannitol	C00024	0.0067	down			(−)
Dodecanedioic acid	C03990	0.0062	down			(−)

^a^ Means the *P* value between CON and DSS groups. Up means DSS group was up-regulated compared with CON group; Down means DSS group was down regulated compared with CON group. ^b^ Means the *P* value between LTD and DSS groups. Up means LTD group was up-regulated compared with DSS group; Down means LTD group was down regulated compared with DSS group. LPC, lysophosphatidylcholine; LPA, lipoprotein A; LPS, lipopolysaccharide.

**Table 3:** The regulation of metabolic pathways in IPEC-J2 cells in CON, DSS and LTD groups (*P* value < 0.01).

No.	Pathways	Total Cmpd.	Biomarkers	-LOG(P)
**Regulation (DSS Group VS. CON Group)**				
1	Pyrimidine metabolism	7	5	1.390822757
2	Glutathione metabolism	6	4	1.023205601
3	beta-Alanine metabolism	3	3	1.452609105
4	Taurine and hypotaurine metabolism	4	3	1.538068701
5	Steroid hormone biosynthesis	2	2	1.366699737
6	Linoleic acid metabolism	2	2	1.366699737
**Regulation (LTD Group VS. DSS Group)**				
1	Pertussis	1	1	1.197280558
2	Phenylalanine metabolism	8	2	1.087752287
3	Aminoacyl-tRNA biosynthesis	5	4	3.080342138
4	Central carbon metabolism in cancer	3	3	2.760981397
5	Protein digestion and absorption	6	4	2.637796477
6	Biosynthesis of amino acids	11	5	2.299502419
7	2-Oxocarboxylic acid metabolism	6	3	1.565706497
8	Mineral absorption	3	2	1.367406194
9	Phenylalanine metabolism	8	3	1.189310988
10	ABC transporters	8	3	1.189310988

**Table 4:** Primers used for gene expression analysis through Real-Time PCR.

Genes	Forward	Reverse
*EGF*	CTGGCTCTGAATGGCCAAGA	TCGCCAACGTAGCCAAAAAC
*CDC2*	GTCGCGGGATAATAAGCTGG	GGAGTGCCCAAAGCTCTGAA
*FGF2*	TTTGGTACCTGCACCCCAAT	GATGTCCCCTTTCCCTACTGT
*Rb*	GTCCGGTTTTTCTCAGGGGAC	ATCCGTGCACTCCTGTTCTG
*P53*	GTCGGCTCTGACTGTACCAC	TTCAGCTCCAAGGCGTCATT
*P21*	ACGTCTCAGGAGGACCATGT	AGAAGATCAGCCGGCGTTTG
*GAPDH*	GGGCATGAACCATGAGAAGT	AAGCAGGGATGATGTTCTGG

## Data Availability

Data are contained within the article.

## Sample Availability

Samples of the compounds are available from the authors.
